# Antiviral Compounds from Myxobacteria

**DOI:** 10.3390/microorganisms6030073

**Published:** 2018-07-19

**Authors:** Lucky S. Mulwa, Marc Stadler

**Affiliations:** 1 Department of Microbial Drugs, Helmholtz Centre for Infection Research and German Centre for Infectio Research (DZIF), Partner Site Hannover/Braunschweig, Inhoffenstrasse 7, 38124 Braunschweig, Germany; luckymulwa@gmail.com; 2 Department of Microbial Strain Collection (MISG), Helmholtz Centre for Infection Research (HZI), Inhoffenstrasse 7, 38124 Braunschweig, Germany

**Keywords:** myxobacteria, antivirals, secondary metabolites, HIV, Ebola, hepatitis viruses

## Abstract

Viral infections including human immunodeficiency virus (HIV), cytomegalovirus (CMV), hepatitis B virus (HBV), and hepatitis C virus (HCV) pose an ongoing threat to human health due to the lack of effective therapeutic agents. The re-emergence of old viral diseases such as the recent Ebola outbreaks in West Africa represents a global public health issue. Drug resistance and toxicity to target cells are the major challenges for the current antiviral agents. Therefore, there is a need for identifying agents with novel modes of action and improved efficacy. Viral-based illnesses are further aggravated by co-infections, such as an HIV patient co-infected with HBV or HCV. The drugs used to treat or manage HIV tend to increase the pathogenesis of HBV and HCV. Hence, novel antiviral drug candidates should ideally have broad-spectrum activity and no negative drug-drug interactions. Myxobacteria are in the focus of this review since they produce numerous structurally and functionally unique bioactive compounds, which have only recently been screened for antiviral effects. This research has already led to some interesting findings, including the discovery of several candidate compounds with broad-spectrum antiviral activity. The present review looks at myxobacteria-derived antiviral secondary metabolites.

## 1. Introduction

Antimicrobial resistance (AMR) threatens the effective treatment, control and management of an increasing range of infections caused by viruses, bacteria, parasites and fungal pathogens [1]. Development of effective therapies to suppress human immunodeficiency virus (HIV) and hepatitis B virus (HBV) has been achieved with great success [2]. However, the medicines are not curative, and therefore more efforts in HIV and HBV drug discovery are directed toward longer-acting therapies or compounds with new mechanisms of action that could potentially lead to a cure or complete eradication of the viruses [2]. In 2010, an estimated 7–20% of people starting antiretroviral therapy (ART) globally had drug-resistant HIV [3]. Some countries have recently reported levels of 15% amongst those starting HIV treatment, and up to 40% among people re-starting therapy [3]. Of great concern are the high levels of viral resistance towards nucleoside reverse transcriptase inhibitors (NRTIs) as recently found in Kenyan children [4]. In 2015, the World Health Organization (WHO) recommended that everyone living with HIV should start on antiretroviral treatment. Hence, increased ART resistance is expected as more people start ART [3]. The United States Food and Drug Administration (FDA) approved anti-HIV drugs are classified into eight classes according to modes of action [5]. The common side effects of Abacavir^®^ and other NRTIs are that they can cause life-threatening side effects, including a serious allergic reaction, a build-up of lactic acid in the blood, and hepatotoxicity [6,7]. In fact, each drug class of the FDA approved anti-HIV drugs has side effects, and some are contra-indicated for co-infected patients with HIV and HBC or HCV [8]. On the other hand, all influenza A viruses circulating in humans were reported to be resistant to Amantadine^®^ and Rimantadine^®^, two essential antivirals for treatment of epidemic and pandemic influenza A. However, the frequency of resistance to Oseltamivir^®^ another antiviral with different mode of action for treating influenza A remains low at 1–2% [3]. Treatment failure of antivirals has been suggested to be caused by the emergence of recombinant viruses, drug resistance, and cell toxicity [9,10]. Compounds with a different mode of action can play an essential role in overcoming AMR. Viral disease such as influenza spreads fast and knows no borders, with the vast masses of people travelling all over the globe due to efficient transport systems. Hence, there is an urgent need for international collaboration to identify new antiviral agents with new modes of action and better efficacy.

Myxobacteria are well-known to be producers of biologically active secondary metabolites with novel carbon skeletons and new modes of action [11,12,13]. Many compounds isolated from myxobacteria have recently been found to have impressive antiviral activity. More so, some have been found to have an unusual broad-spectrum antiviral activity [11,12,14].

## 2. Myxobacteria

**Figure 1:**
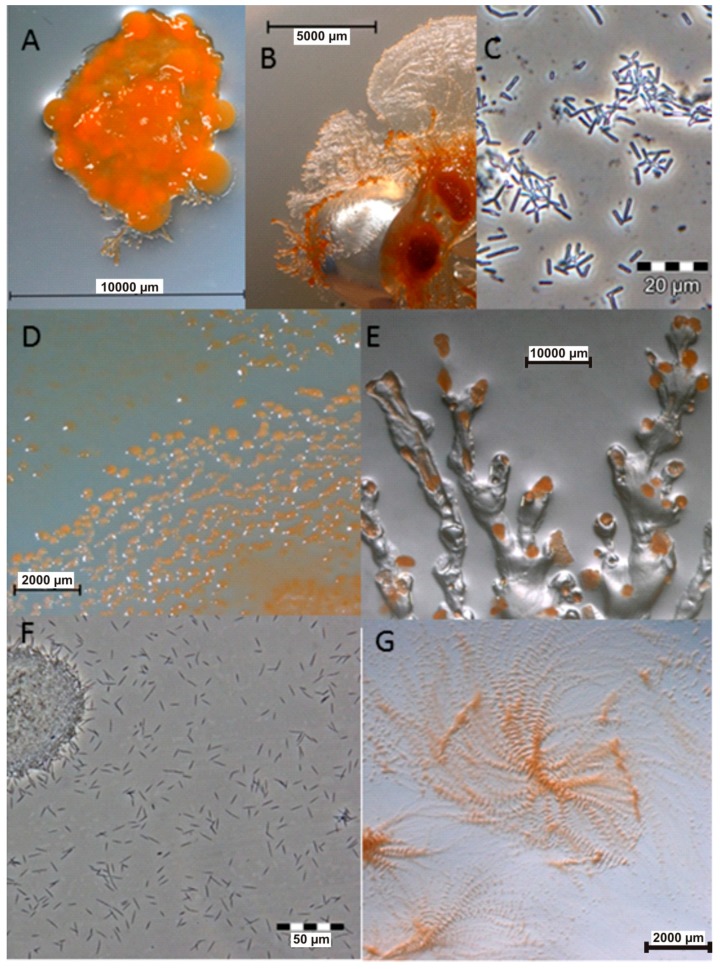
Images of myxobacteria. (**A**–**C**): *Sorangium cellulosum*; (**A**): Fruiting bodies; (**B**): Swarming on agar plate; (**C**): Cells from the liquid medium under the light microscope. (**D**–**E**): Images of the producers ofthiangazole (**7**), phenalamide A_1_ (**8**) and phenoxan (**9**), from agar plates; (**D**): *Myxococcus stipitatus*; (**E**): *Polyangium* species; (**F**–**G**): *Angiococcus disciformis* (strain An d30) producer of myxochelins; (**F**): culture under the light microscope from liquid media; (**G**): culture on agar plate. Images provided by Joachim Wink (HZI Braunschweig).

Myxobacteria are δ-proteobacteria belonging to the order *Myxococcales*. They are rod-shaped, Gram-negative bacteria that exhibit gliding motility and swarm on solid surfaces. Under nutrient-limiting conditions, they form species-specific fruiting bodies (Figure 1) [15]. Within these fruiting bodies, some vegetative cells convert to myxospores, which are desiccation-resistant and can survive over decades. Under appropriate conditions the spores germinate [16]. These soil-dwelling microorganisms have also been isolated from other habitats such as the bark of trees, oceans, freshwater lakes, and herbivore dung [15,17]. Myxobacteria have also been isolated from extreme environments such as desert soils [18]. Numerous unique classes of secondary metabolites have been isolated from myxobacteria, the majority of which are biogenetically derived from polyketide synthases (PKSs) and non-ribosomal peptide synthetases (NPRSs) or a hybrid of PKSs and NPRSs [12,15,19,20]. PKSs and NPRSs are enzymatic “assembly lines” of complex multi-step biosynthetic pathways for making compounds by catalysing the stepwise condensation of a starter unit with small monomeric building blocks [19]. The ability to produce unique metabolites is conferred by the creative biosynthetic pathways and the large genome of 9–14 Mb [21], consistent with the strengthening correlation between genome size and the extent of secondary metabolites produced [21,22,23,24,25]. In fact, the sequenced genomes of myxobacteria are the largest yet known from any bacterium [20,21,22,23,25]. In the last 35 years, over 100 new carbon skeleton secondary metabolites, with over 600 analogues, have been isolated from over 9000 strains of myxobacteria [12]. The metabolites exhibited antifungal, antibacterial, antimalarial, antitumor, and anti-immunomodulatory properties some with novel modes of action and have been reviewed extensively [12,20,26]. Microorganisms are valuable as producers of bioactive metabolites because they can be cultivated in bioreactors from as little as below 5 mL to large scales of over 100,000 L, making the production of natural products independent of season, locality, or climate [15]. Furthermore, conditions in a bioreactor are controllable to optimise production of the desired outcome. Particularly important as illustrated by Zeeck et al., 2002 in the ‘OSMAC’ (One Strain-Many Compounds) approach, which revealed that microorganisms do not exhaust their potential for producing metabolites under standard laboratory conditions [27].

## 3. Secondary Metabolites from Myxobacteria with Antiviral Activity

### 3.1. Human Immunodeficiency Virus (HIV)

HIV is a lentivirus of the Retroviridae family. HIV targets immune cells, and reverse transcribes its single-stranded RNA (ssRNA) genome, integrating into the host chromosomal DNA. The virus uses high antigenic diversity and multiple mechanisms to avert recognition by the human immune system thus posing a challenge to host defences and treatment [28].

Various assays have been developed and used to identify molecules with anti-HIV activity. Some of the assays includes structure-based design of a small molecule CD4-antagonist with broad spectrum anti-HIV-1 activity [29], structure-based identification of small molecule antiviral compounds targeted to the gp41 core structure of the human immunodeficiency virus type 1 [30], and identification of HIV inhibitors by high-throughput (HTP) two-step infectivity assay [31]. The HTP assay has been used on myxobacterial-derived molecules with success due to the ability to screen a large number of molecules in a short period.

**Figure 2:**
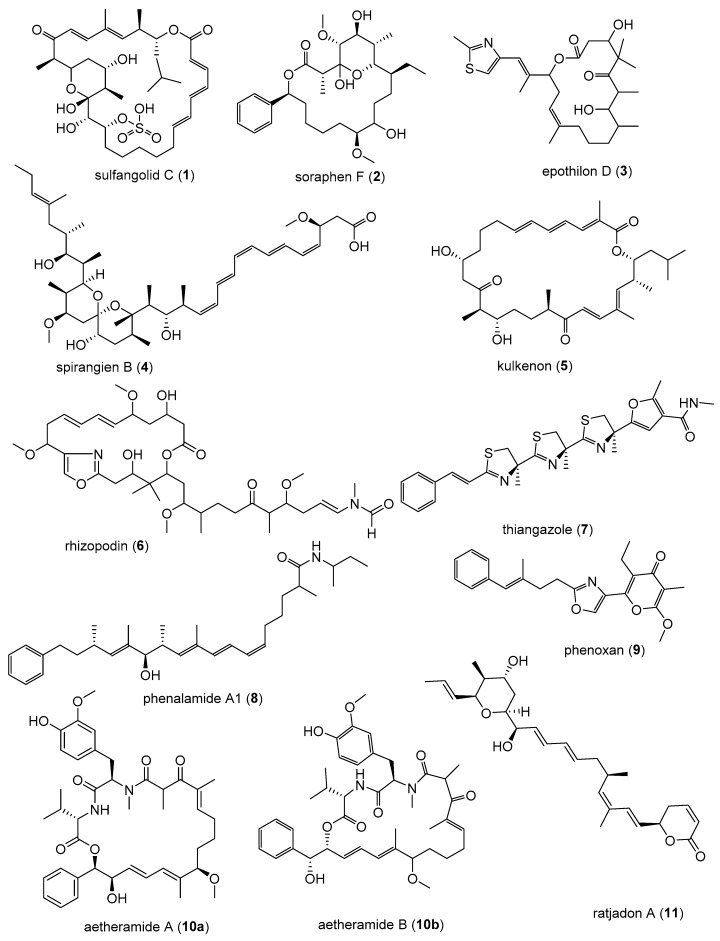
Myxobacterial-derived compounds with activity against human immunodeficiency virus (HIV).

Sulfangolids, the first sulfate esters containing a series of secondary metabolites produced by several strains of *Sorangium cellulosum*, were isolated together with the structurally related macrolide kulkenon (**5**) [32]. Sulfangolid C (**1**), soraphen F (**2**), epothilon D (**3**), and spirangien B (**4**), showed impressive activity, with EC_50_ values in the nM range with a selectivity index value greater than 15 (SI > 15) in the high-throughput two-step infectivity assay [31]. Despite the impressive antiviral activity of **5**, the SI is low because of toxicity. A search in the SciFinder database revealed dozens of analogues of soraphen and epothilone [33]. It may be promising to screen the large number of analogues in the soraphen and epothilone families to attempt to encounter more potent representatives with better SI values. The soraphens have been reported as acetyl-CoA carboxylate transferase inhibitors [34], while the epothilones stabilise microtubuli of macrophages in a similar manner as Taxol^®^ without showing taxol-like endotoxin activity [35,36]. In fact, the FDA-approved anticancer drug, Ixabepilone^®^, is an epothilone B derivative [37]. Metabolites **3** and **4** are reported to decelerate the phosphorylation and degradation of inhibitor of kappa Bα (IkBα) [36,38]. The compounds identified as preliminary hits for anti-HIV included **1**–**5** (Table 1, Figure 2) [31]. Rhizopodin (**6**), from *Myxococcus stipitatus* was identified as interesting in the two step HTP assay, likely because of its mode of action [31]. HIV cell-to-cell transmission is the primary route of HIV infection in naive cells in vivo. Actin filaments are known to be essential for virological synapse formation, therefore, virus synapses are interfered by **6**, which is a known actin inhibitor. Disorazol, tubulysin and stigmatellin variants were also reported to have mild anti-HIV activity [31]. Thiangazole (**7**), phenalamide A_1_ (**8**), and phenoxan (**9**) isolated from two strains of *Polyangium* sp. and *Myxococcus stipitatus* strain Mx s40 were reported to have anti-HIV activity (Figure 2) [39]. They all revealed high activity by suppressing HIV-1-mediated cell death in the MT-4 cell assay with EC_50_ values of **9** and **8** in the nanomolar range, whereas thiangazole (**7**) had an impressive EC_50_ value in the picomolar range, making it a possible lead compound for anti-HIV therapy (Table 2, Figure 2) [39]. In another assay involving measuring ATP levels as a parameter of cell viability of TZM-bl cells aetheramide A (**10a**) and aetheramide B (**10b**) isolated from the recently described genus *Aetherobacter*, inhibited HIV-1 infection with IC_50_ value of 0.015 and 0.018 µM, respectively [40,41,42]. Concurrently, the aetheramides were reported to be moderately antifungal and cytotoxic [41]. The chemical structures of **10a** and **10b** are rare, containing a polyketide moiety and two amino acid residues, thus forming a new class of antivirals [40,41,42]. This discovery of new antivirals from the recently described myxobacteria genus, *Aetherobacter*, represents an example of the enormous biosynthetic capabilities of myxobacteria and their importance to drug discovery efforts [42]. Ratjadon A (**11**), an α-pyrone metabolite isolated from *Sorangium cellulosum* (strain Soce 360), was reported to inhibit HIV infection by blocking the Rev/CRM1-mediated nuclear export pathway [43,44]. The CRM1-Rev complex is an attractive target for the development of new antiviral drugs because the nuclear export of unspliced and partially spliced HIV-1 mRNA is mediated by the recognition of a leucine-rich nuclear export signal (NES) in the HIV Rev protein by the host protein CRM1/Exportin1 [44]. Despite **11** being reported to exhibit a strong anti-HIV activity, it has a low selectivity due to toxic effect. The low SI value limits the potential use of **11** as a therapeutic drug. More studies with derivatives of **11** need to be done. It is important to observe that different assays were used to screen for anti-HIV compounds. There is a need therefore for a standardised method to be able to adequately compare the anti-HIV activity of those compounds that have been identified as preliminary hits. Equally important is an evaluation of the mechanism of action on viruses in comparison to the mechanism of action on bacteria or fungi. Investigation for synergism between the identified anti-HIV compounds for possible use at a lower concentration to improve the selectivity index of the metabolites should be looked into in the future. Even the active metabolites that cannot realistically be further developed as drug candidates because they are too toxic, could be used as biochemical tools to attain a better understanding of the invasion mechanism of HIV, or for development of synthetic analogues that mimic these compounds without causing toxicity.

### 3.2. Human Cytomegalovirus (HCMV)

**Figure 3:**
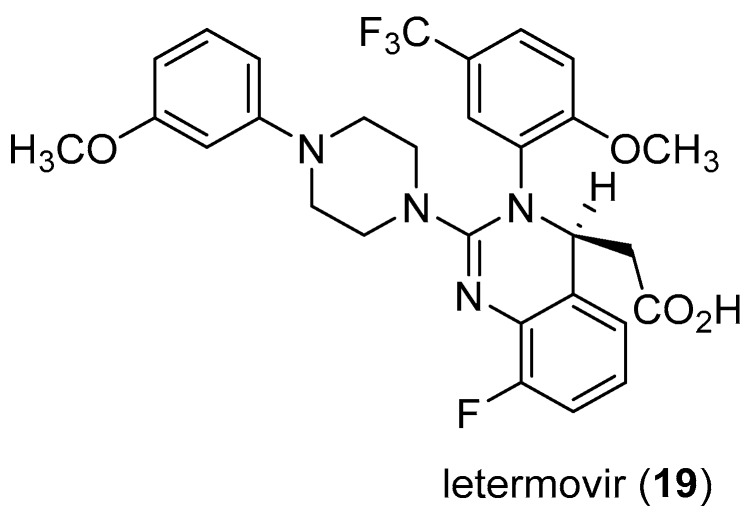
PREVYMIS™ (letermovir) a recently (2017) FDA-approved drug for the prevention of *Human cytomegalovirus* (HCMV) infection and disease in organ transplant patients.

HCMV belongs to the β-herpesvirus family, with a high prevalence, infecting up to 80% of the general population usually asymptomatic in healthy people [45]. Diseases associated with HCMV include glandular fever and pneumonia. HCMV is also an important pathogen in organ transplant patients responsible for significant morbidity and mortality in organ transplant recipients, and a major cause of disease in patients with HIV infection [46]. HCMV infections in newborns may result in hearing loss, mental retardation and palsy [47]. The available FDA-approved therapeutic options for HCMV infection include ganciclovir, foscarnet, cidoforvir, and fomivirsen [48]. These drugs have different mechanisms of actions or applications, and represent the successes that had been made against the challenges of HCMV [48,49,50]. Several anti-HCMV drugs were reported to have low potency, poor oral bioavailability, and adverse side effects [50]. Moreover, drug resistance strains were reported to emerge [51]. Hence, there has been a renewed interest in search of new inhibitors of HCVM [50,51]. Of greater concern is the increase in the number of people living with transplanted organs, and the increase in HIV infected people [52]. Technological advancements have enabled organ transplants to be more accessible while the increase in HIV-infected individuals is due to new retroviral therapies that have converted HIV infection to chronic disease as infected people live longer, leading to increased cases of HCMV infection [52]. In 2011, the first case of HCMV-treated with AIC246, a novel anti-CMV compound that targets the viral terminase complex and remains active against virus resistant to DNA polymerase inheritors was reported [53], which represents a good example of the renewed interest for HCMV inhibitors [53]. Almost 60 patents claiming novel agents for the treatment of HCMV were launched from January 1996 to 2000, but so far none of these projects has led to the approval of an anti-HCMV drug [49]. However, the recent FDA approval of letermovir (Figure 3), providing a long-awaited alternative for preventing cytomegalovirus infection in allogeneic hematopoietic stem cell transplant recipients [54] is very encouraging.

A class of myxobacterial compounds, myxochelin, belonging to a larger group of natural products, siderophores, were isolated from several strains of myxobacteria [53,55]. Siderophores are secondary metabolites produced by some microorganisms under iron-limiting conditions, and enhance the uptake of iron [56]. Other siderophores isolated from myxobacteria includes nannochelins and hylachelins. Various studies have revealed myxochelins to be potent antitumour agents [55,57,58,59]. The antitumour activity was demonstrated to be caused by inhibition of human 5-lipoxygenase (5-LO) [58]. Surprisingly the inhibition of 5-LO by myxochelins was found not correlating with the iron affinities [58]. The enzyme 5-LO is responsible for the catalysis of two initial steps in the biosynthesis of leukotriens, starting from arachidonic acid [58]. Leukotriens are well-known mediators of a variety of allergic reactions such as inflammatory, rheumatic arthritis, allergic rhinitis and cardiovascular diseases [58]. Importantly, 5-LO pathways were associated with cancer proliferation, hence explaining the observed strong anticancer activity of myxochelin [58,60]. Nannochelins are reported to have no significant antimicrobial activity [24].

**Figure 4:**
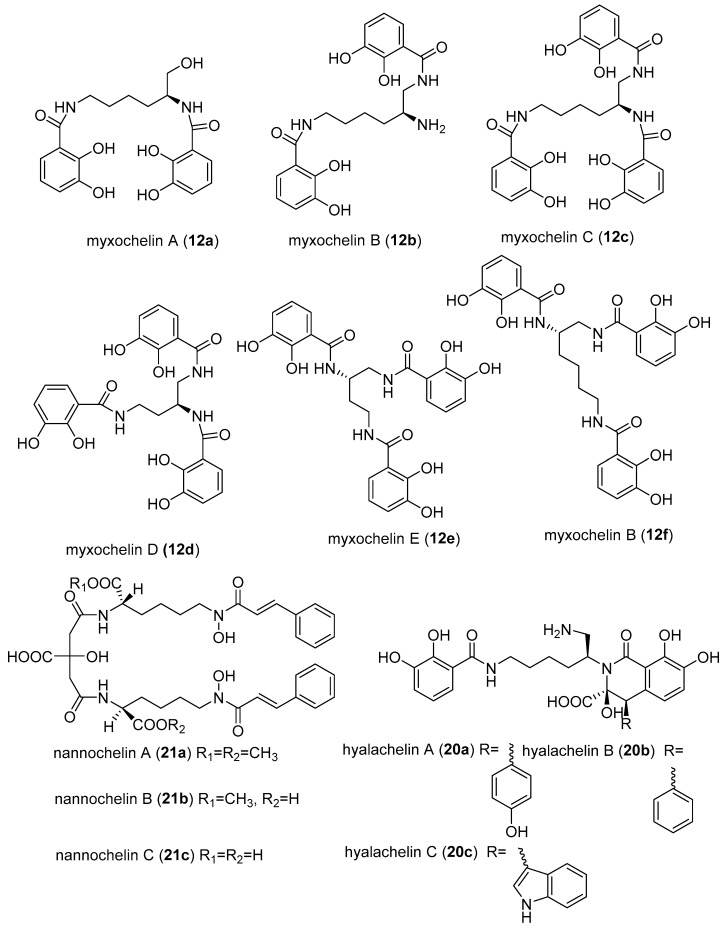
Myxobacterial-derived siderophores.

Myxochelin A (**12a**) was initially isolated from the culture broth of *Angiococcus disciformis* (strain An d30). Later on, myxochelins B (**12b**), C (**12c**), D (**12d**), E (**12e**), and F (**12f**) were isolated and also synthesised (Figure 4) [60,61]. The corresponding biosynthetic gene clusters have been identified in *Stigmatella aurantiaca* (Sga 15), and *Sorangium cellulosum* (Soce 56) [62]. Additional siderophores have been isolated from *Nannocystis exedens* (**21a**–**21c**) and *Hyalangium minutum* (**20a**–**20c**) [63,64]. Myxochelin C (**12c**) inhibited HCMV with an IC_50_ value of 0.7 µg/mL [46,53]. It could in future become feasible to test others among the over 500 different siderophores that are known to science [65]. In particular, the known myxobacterial-derived siderophores, such as nannochelins (**21a**–**21c**), hylachelins (**20a**–**20c**), and all the other myxochelin analogues (Figure 4) should be screened for various antiviral activities, especially anti-HCMV, and should be studied for structure activity relationship for possible discovery of more potent antivirals.

### 3.3. Ebola Virus Disease (EVD)

Ebola haemorrhagic fever is caused by the Ebola virus (EBOV), a single stranded RNA enveloped virus belonging to the family *Filoviridae*. EVD first appeared in 1976 in two simultaneous outbreaks, one in Nzara, South Sudan, and the other in Yambuku, the Democratic Republic of Congo. The latter occurred in a village near the Ebola River, from which the disease takes its name [65,66]. EVD case fatality rate is around 50%, with different cases from 25% to 90% fatality in past outbreaks reported [65]. Furthermore, EBOV is known to persist in immune-privileged sites, such as testicles, inside of the eye, and central nervous system, and in some people who have recovered from EVD [65]. The effect of the persistence is yet to be known [66].

**Figure 5:**
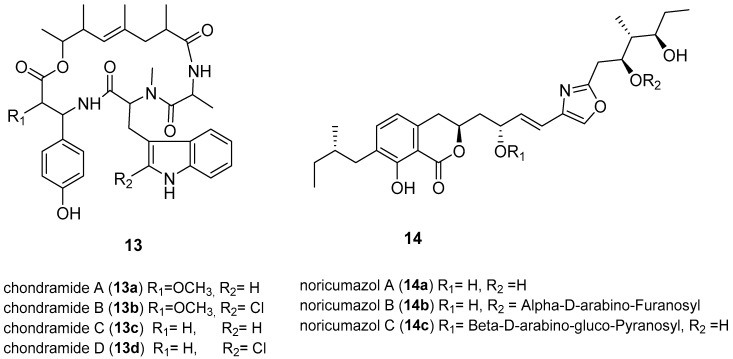
Structures of myxobacterial-derived compounds with activity against Ebola virus (EBOV). Chondramides (**13a**–**d**) are known to be actin inhibitors, while noricumazols (**14a**–**c**) are known potassium channel inhibitors [70,71].

The re-emergence of Ebola occurred in West African countries causing 11,308 deaths leading to the WHO on 8 August 2014, declaring the epidemic to be an international public health emergency [66]. An experimental Ebola vaccine called rVSV-ZEBOV has been reported to show high protection against EVD [67]. No drug or licensed vaccine currently exists; hence there is an urgent need for drugs that inhibit entry or multiplication of EDV [62,67]. Developing an assay to test compounds for anti-EBOV poses a significant challenge because of the cost of equipment for the high risk, Biosafety S4 organism, involved [66]. However, various metabolites from myxobacteria were screened for EBOV inhibition by an assay with a surrogate system using Ebola envelope glycoprotein GP-pseudotyped lentiviral vectors (Figure 5) [67]. GP-pseudotype lentiviral vectors were used as tools to investigate the entry process of the viruses, enabling studies without the need of using the native Ebola virus reducing the safety level from the highest level 4 to level 2 [67]. The same analysis was conducted with vesicular stomatisis virus (VSV)-G-pseudotyped vectors to determine the EBOV-specificity of the inhibitory function of the compounds. Chondramides (**13a**–**13d**) were reported to inhibit EBOV-GP-mediated transduction with impressive IC_50_ values of 24–42 nM. The VSV-G-mediated transduction was less efficient, with an IC_50_ value of 55–111 nM [66]. Chondramides (**13a**–**13d**), a class of compounds known to interfere with actin, were isolated from a myxobacterium belonging to the genus *Chondromyces* [68]. Members of the genus *Chondromyces* belong to those myxobacteria known to synthesise two or more chemically unrelated secondary metabolites with different mechanisms of action [69]. Other promising hits were the noricumazoles, a family of polyketides from *Sorangium cellulosum*. Noricumazole A (**14a**) was found to inhibit EBOV-GP with an IC_50_ value of 0.33 µM. In fact, **14a** was found to be EBOV-GP specific and showed no significant inhibition against (VSV)-G-pseudotyped vectors [67]. Noricumazoles are known to be potassium channel blockers with **14a** known to be highly toxic while the derivatives, **14b** and **14c**, are equally active with lower toxicity [67,70]. The screening of myxobacterial natural compounds library resulted in the identification of inhibitors of EBOV-GP pseudotyped vectors, chondramides and noricumazole, whose mechanism of action is actin-stabilising and the channel blockers respectively [67]. These metabolites will give insights into the EBOV infection mechanism, rather than being used as drugs, because the modes of actions are expected to have side effects. However, the lower toxicity of **14b** and **14c**, which are derivatives of **14a**, is exciting and qualifies **14a** to be considered as a lead structure for the development of EBOV inhibitors.

### 3.4. Hepatitis C Virus

HCV is an enveloped, single-stranded RNA virus with positive polarity (ss (+) RNA). HCV is transmitted by blood-to-blood contacts, such as through intravenous injections, blood transfusion, and various exposures to blood contaminants. It can also be transmitted by contact with bodily fluids including saliva or semen of an infected person [72]. By 2015, there were 71 million people infected with HCV globally [73]. HCV and hepatitis B virus (HBV) infection are the major causes of hepatocellular carcinoma (HCC), associated with cirrhosis [74]. Currently, no products are available to prevent HCV infection. There are some drugs available that can cure HCV infection [75]. However, treatment is complicated by HIV-HCV/HBC co-infections with drug-drug interactions between anti-HIV and anti-HCV drugs, resulting in serious side effects and can lead to the death of patient [8]. The discovery of broad-spectrum antivirals may play an essential role in overcoming this challenge. 

**Figure 6:**
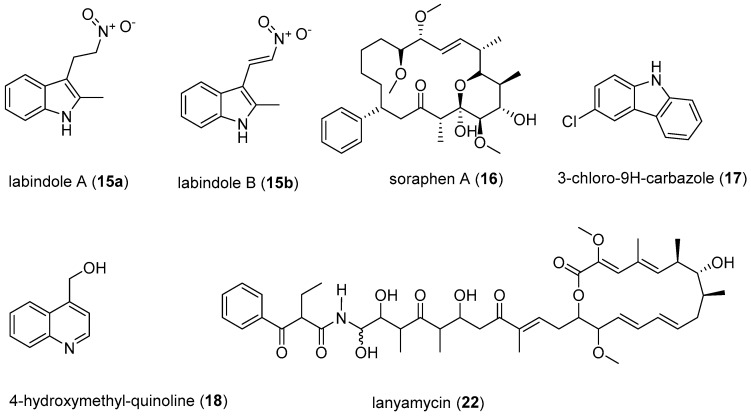
Compounds with activity against Hepatitis C Virus.

The recently isolated compounds from *Labilithrix luteola*, labindoles A (**15a**), and B (**15b**) have been reported to have moderately inhibited HCV (Figure 6) [75]. Interestingly the labindoles were said to have no cytotoxicity, anti-bacterial or antifungal activities. 3-chloro-9H-carbazole (**17**) and 4-hydroxymethyl-quinoline (**18**) also isolated from *Labilithrix luteola* were reported to have a strong inhibition of HCV [75]. Soraphens are a family of polyketide-derived macrolactones comprising over 50 metabolites known for strong antifungal activity [76]. However, recent studies have suggested that soraphen A (**16**) inhibits HCV replication in HCV cell culture models expressing subgenomic and full-length replicons as well as a cell culture-adapted virus with an IC_50_ value of 5 nM [11,77]. The HCV assay involved the development of subgenomic replicons that replicate autonomously in the human hepatoma cell line Huh-7 to be able to screen for anti-HCV activity. The subgenomic replicons are genetic materials from HCV, which represent the actual invasion and replication of HCV on the liver cells [75]. Furthermore, **16** is known to depolymerise the acetyl-CoA carboxylase (ACC) complexes into less active dimers [77]. The mechanism of action of **16** is a valuable probe to study the roles of ACC polymerisation and enzymatic activity in viral pathogenesis [77]. Various minor structure alterations of **16** did not affect the antiviral activity [11]. Owing to the fact that soraphens inhibit both HIV and HCV, it has been proposed that the broad-spectrum activity of **16** could be due to targeting commonly used host factors or pathways necessary for viral replication [78]. Another recently isolated myxobacteria-derived secondary metabolite, lanyamycin (**22**) from *Sorangium cellulosum* (strain Soce 481) moderately inhibited HCV with IC_50_ value of 11.8 µM [78]. The macrolide, **22**, is closely related to the bafilomycins, a class of secondary metabolites from actinobacteria [79]. Interestingly bafilomycin A was previously shown to possess good activity (IC_50_ value of 0.1 nM) against influenza A virus, which is below its cytotoxic levels [80]. Screening of **22** for activity against influenza A virus and other pathogenic viruses, and studying the mode of action would be interesting.

## 4. Conclusions

The reported antivirals discoveries call for more screening of myxobacterial-derived compounds, especially against other medically important viruses. Myxobacteria have demonstrated to be creative producers of molecules that can have valuable applications as possible lead structures for the development of antiviral drugs. Some broad-spectrum antivirals such as soraphen A could be of interest for the possibility of treating co-infection cases. Further, the possibility of using these myxobacteria derived secondary metabolites for treating both opportunistic infections and the HIV could be explored. The challenge of some compounds being toxic can be approached by structure modification for possibility of reducing toxicity while maintaining efficacy, as observed with the analogues of soraphen and noricumazoles. Moreover, some metabolites found to have potent antiviral activity may not be used as drugs themselves, due to toxicity, but they can serve as excellent tools to study and understand the viral invasion. This valuable information can be used to select other metabolites with similar mechanisms of action or structural modification of the compounds, to reduce their toxicity without substantially altering the activity. This ability of myxobacteria to produce such a vast number of secondary metabolites is most likely brought about by many years of evolution to adapt to survival in an ecological condition of competitive existence in the presence of competitors and invaders such as fungi, bacteriophages, and other bacteria. A recent comprehensive study involving molecular phylogeny with HPLC-MS profiling has revealed an unparalleled diversity of metabolites, along with strong correlations of the metabolite production to the phylogenetic position of the corresponding producer organisms [81]. Therefore, it appears promising to isolate more myxobacteria that represent novel genera and species from unexplored environments and screen them systematically for the production of further unique compounds with antiviral activities.

## Figures and Tables

**Table 1:** Preliminary anti-HIV hits from a high-throughput two-step infectivity assay [31].

Compound	MW ^1^	EC_50_ (µM)	CC_50_ (µM)	SI **
Nevirapine *	266	0.07	81.8	>10^3^
sulfangolid C (**1**)	682	0.41	8.18	20.2
soraphen F (**2**)	522	0.30	5.02	16.5
epothilon D (**3**)	491	0.0005	0.012	24.4
spirangien B (**4**)	717	0.007	0.35	52
kulkenon (**5**)	734	0.07	0.36	5.3

* Control, ^1^ Molecular weight, EC_50_: effective concentration; CC_50_: cytotoxic concentration; ** Selectivity Index = CC_50_/EC_50_. EC_50_ is the concentration of a drug or metabolite which induces a response halfway between the baseline and maximum after a specified exposure time or gives the desired effect to 50% of test subjects. While SI is a comparison of the amount of a drug or metabolite that causes the desired effect to the amount that causes death or toxicity. Metabolites with a low EC_50_ and a high SI values are good drug candidates.

**Table 2:** Anti-HIV-1 activities of compounds derived from myxobacteria by MT-4 cell assay [40,41].

Compound	MW ^1^	Tx ^2^ (nM)	AE ^3^ (µM)	SI **
AZT *	267	250,000	25	10^4^
thiangazole (**7**)	539	>4700	0.0047	>10^6^
phenalamide A_1_ (**8**)	491	102,000	1.02	10^5^
Phenoxan (**9**)	379	>6600	6.6	>10^3^

* azidothymidine Control, ^1^ Molecular weight, ^2^ Toxicity (Tx) is the lowest toxic concentration (nM) of the compound in the MT-4 cell assay, ^3^ Antiviral efficacy (AE) is given as the lowest effective concentration (µM) of the compound at which 100% prevention of the virus-mediated cytopathogenicity was observed in the MT-4 cell assay, ** Selectivity Index = Tx (nM)/AE (µM).
